# Transvitreal and subretinal fibrinoid reaction following diabetic vitrectomy

**DOI:** 10.1016/j.ajoc.2022.101594

**Published:** 2022-05-24

**Authors:** Andrew J. Nelson, Neesurg S. Mehta, Jorge R. Ochoa, Caesar Luo, Kareem Moussa

**Affiliations:** a UC Davis Eye Center, University of California Davis, Sacramento, CA, USA; b Bay Area Retina Associates, Oakland, CA, USA

**Keywords:** Proliferative diabetic retinopathy, Vitrectomy, Surgical complications

## Abstract

**Purpose:**

To describe an unexpected and atypical transvitreal and subretinal fibrinoid reaction following vitrectomy for non-clearing vitreous hemorrhage due to proliferative diabetic retinopathy.

**Observations:**

A 58-year-old woman with bilateral proliferative diabetic retinopathy and non-clearing vitreous hemorrhage underwent combined phacoemulsification, intraocular lens implantation and vitrectomy in the right eye. Two months later she underwent staged phacoemulsification with intraocular lens implantation followed by vitrectomy in the left eye. The early postoperative course of each eye was complicated by choroidal effusion and submacular fibrinoid material on optical coherence tomography. The left eye also developed transvitreal fibrinoid bands.

**Conclusions and importance:**

Subretinal fibrin accumulation is a rare complication of diabetic vitrectomy. Optical coherence tomography (OCT) in the early post-operative period may assist in the recognition of this complication.

## 1 Introduction

Pars plana vitrectomy is commonly performed for non-clearing vitreous hemorrhage in patients with proliferative diabetic retinopathy (PDR), and generally provides a significant improvement in visual acuity with low incidence of short-term complications.^1^ Although subretinal fluid^2^^,^^3^ and choroidal effusion^4^ can be seen following diabetic vitrectomy with endolaser panretinal photocoagulation, the appearance of transvitreal fibrinoid bands during the early postoperative period is a rare complication. Sebestyen first described “fibrinoid syndrome” after a series of diabetic patients developed transvitreal fibrinoid strands following vitrectomy, which ultimately progressed to tractional retinal detachment and neovascular glaucoma.^5^ More recently, Luo et al. described a similar phenomenon in a series of 7 patients, however the transvitreal fibrinoid bands resolved within a week with postoperative topical medications.^6^ Here, we report a case of a patient with fibrinoid material in the vitreous cavity and subretinal space following diabetic vitrectomy.

## 2 Case report

A 58-year-old Hispanic woman was referred to our clinic for poor vision in both eyes for 6 months. She had a history of type 2 diabetes mellitus a hemoglobin A1C of 13.1% at presentation and end-stage renal disease requiring dialysis. Visual acuity was hand motion in the right eye (OD) and count fingers at one foot in the left eye (OS). Intraocular pressures (IOP) were normal and exam was notable for cataract and dense vitreous hemorrhage in both eyes with no view to the posterior pole. No neovascularization of the iris or angle were noted in either eye. The retina was attached in both eyes on B-scan ultrasonography with a measured axial length of 22.4 mm OD and 22.1 mm OS. Scleral thickness measured at the posterior pole was 0.9 mm OD and 1.1 mm OS. She underwent cataract extraction with intraocular lens implantation, vitrectomy, epiretinal membrane peel, endolaser, partial fluid-air exchange, intravitreal bevacizumab injection (1.25mg/0.05mL), and posterior subtenon's triamcinolone acetonide injection (20mg/0.5mL) in the right eye. Regressed neovascularization of the disc (NVD) was noted intraoperatively and a preretinal membrane was peeled off the disc. Duration of surgery was 90 minutes.

**Fig. 1:**
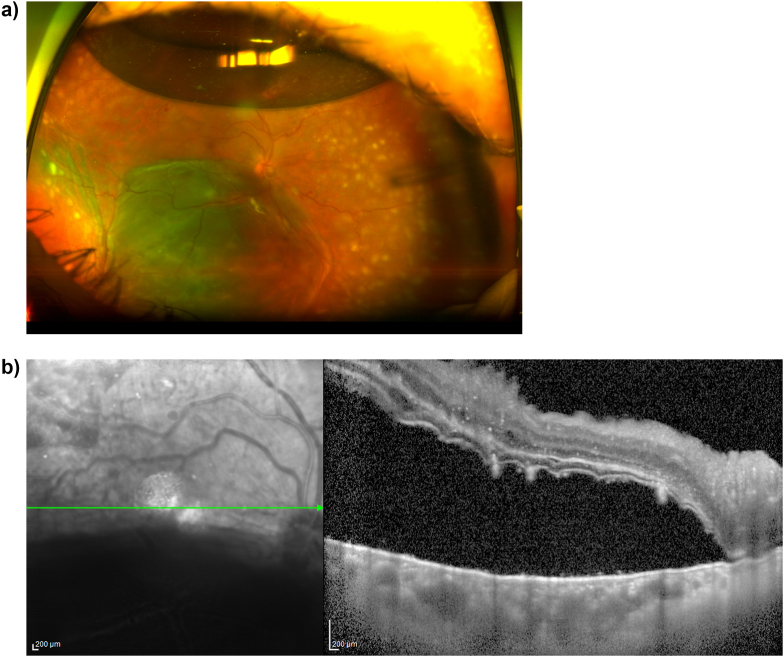
a) Ultrawide field color fundus image of the right eye on postoperative day 4 demonstrates peripheral choroidal effusion and inferior serous retinal detachment. b) Optical coherence tomography of the macula of the right eye on postoperative day 4 demonstrates submacular fluid and hyperreflective material. (For interpretation of the references to color in this figure legend, the reader is referred to the Web version of this article.)

Postoperative day (POD) 1 exam was notable for choroidal effusion and an inferior serous retinal detachment. IOP was normal (17 mmHg) and slit lamp exam was notable for 1+ cell in the anterior chamber. On POD 4, optical coherence tomography (OCT) revealed submacular fluid and fibrinoid material (Fig. 1). She was treated with a sub-tenon's injection of triamcinolone acetonide (40mg/1mL) with near-complete resolution of the submacular fluid and fibrinoid material two weeks later.

**Fig. 2:**
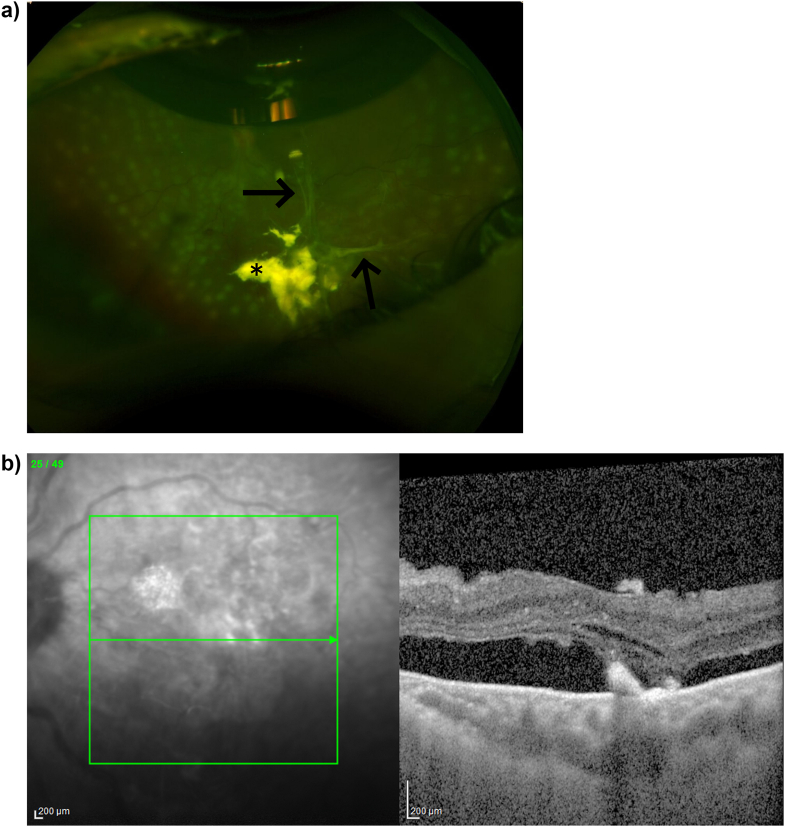
a) Ultrawide field color fundus image of the left eye on postoperative day 4 demonstrates a superior air bubble, submacular fluid, inferior transvitreal fibrinoid bands, and the presence of intravitreal triamcinolone immediately following intravitreal triamcinolone acetonide injection. Asterisk denotes the triamcinolone acetonide and the arrow points to the transvitreal fibrinoid strands. b) Optical coherence tomography of the macula of the left eye on postoperative day 4 demonstrates subretinal fluid and subretinal hyperreflective material. (For interpretation of the references to color in this figure legend, the reader is referred to the Web version of this article.)

The left eye underwent uneventful cataract surgery followed by pars plana vitrectomy, endolaser, epiretinal membrane peel, partial fluid-air exchange, and intravitreal bevacizumab injection (1.25mg/0.5mL) 1 month later in a staged approach. Similar to the right eye, regressed NVD was noted intraoperatively and a preretinal membrane was peeled off the disc. Duration of surgery was 90 minutes. POD 1 exam was again notable for choroidal effusion and inferior serous subretinal fluid, in addition to transvitreal fibrinoid bands. Intraocular pressure was 14 mmHg and llit lamp exam was notable for 1+ cell in the anterior chamber. Imaging captured on POD4 showed these fibrinoid bands, and OCT revealed submacular fluid and fibrinoid material (Fig. 2). She was treated with intravitreal triamcinolone acetonide (2mg/0.05mL) with near-complete resolution of the submacular fluid and fibrinoid material two weeks later.

**Fig. 3:**
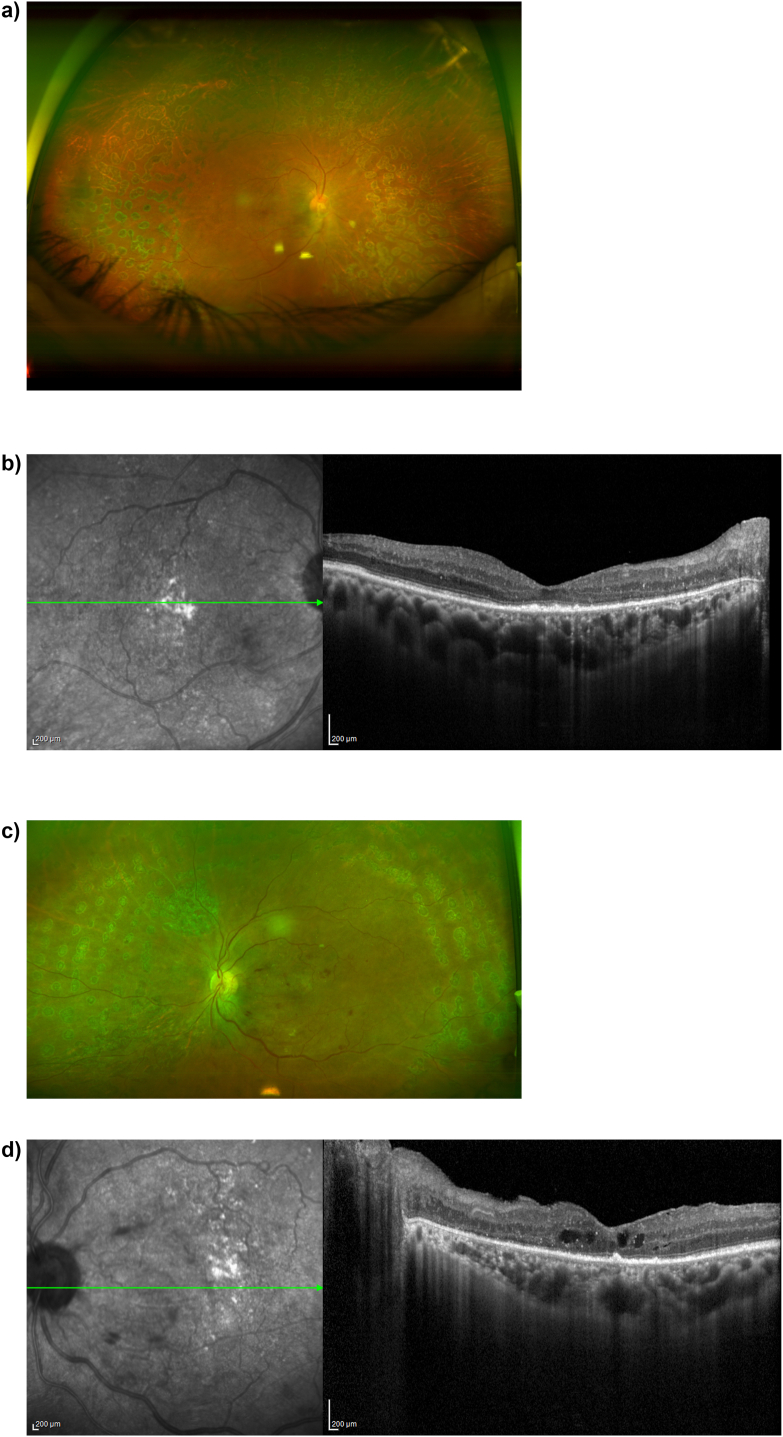
a) Ultrawide field color fundus image of the right eye 6 months after surgery. b) Optical coherence tomography of the macula of the right eye 6 months after surgery demonstrates mild extrafoveal intraretinal fluid and patchy ellipsoid zone atrophy. c) Ultrawide field color fundus image of the left eye 2.5 months after surgery. d) Optical coherence tomography of the macula of the left eye 2.5 months after surgery demonstrates center-involving intraretinal fluid. (For interpretation of the references to color in this figure legend, the reader is referred to the Web version of this article.)

6 months after surgery on the right eye and 2.5 months after surgery on the left eye, visual acuity was 20/100 OD and counting fingers OS, and the retina was attached in both eyes (Fig. 3). OCT showed mild extrafoveal intraretinal fluid and patchy ellipsoid zone loss OD, and center involving intraretinal fluid OS (Fig. 3).

## 3 Discussion

We report a case of bilateral choroidal effusion, serous retinal detachment, and submacular fibrinoid material following diabetic vitrectomy, as well as transvitreal fibrinoid strands in the left eye. Choroidal effusion is a common complication of laser treatment for PDR, and the incidence is correlated with the number and size of laser spots applied.^4^^,^^7^ Laser treatment is thought to increase the vascular permeability in the choriocapillaris, resulting in transudation of fluid into the supraciliary space. Local corticosteroid injections have been shown to reduce the incidence of this complication.^8^

Our case shares similar characteristics to cases that have been reported previously. These cases have been described as pseudoendophthalmitis,^6^ vitreous web,^9^ or fibrinoid syndrome,^5^ and were characterized by the appearance of transvitreal fibrinoid bands without pain or significant anterior segment inflammation following diabetic vitrectomy. In Sebestyen's case series, fibrinoid syndrome appeared to have a poor prognosis, as all cases progressed to tractional retinal detachment, and nine out of fifteen cases resulted in no light perception vision.^5^ The other six cases appeared to respond well to high doses of topical and systemic corticosteroid. Performing cataract extraction or scleral buckling in addition to vitrectomy appeared to be risk factors. More recently, Luo et al. reported eight cases of post-vitrectomy transvitreal fibrinoid band formation, and each of these cases resolved within 2 weeks with topical corticosteroid treatment alone.^6^ Submacular fibrinoid material was not reported in these cases. Advancements in surgical techniques and modifications to postoperative management may explain the more benign course of the fibrinoid reaction in more recent reports.

The pathophysiology of fibrinoid reactions after diabetic vitrectomy is poorly understood. Sebestyen hypothesized that vitrectomy and endolaser may cause further insult to pre-existing damage to the blood-retinal barrier from diabetic eye disease, resulting in exudation of plasma lipid and proteins into the vitreous cavity and activating fibrin. Luo et al. also postulated that the air-fluid meniscus may provide an interface which facilitates the propagation of fibrin. In our case, we suspect the exuberant inflammatory response consisting of choroidal effusion, serous retinal detachment, and submacular fibrinoid material in both eyes, and the transvitreal fibrinoid bands in the left eye, may be due at least in part to the application of complete panretinal photocoagulation intraoperatively in an eye with a significant amount of ischemia and a compromised blood-retinal barrier. Alternative considerations include idiopathic uveal effusion syndrome, although this is less likely as this condition is usually associated with short axial lengths, as well as central serous chorioretinopathy (CSC), which is unlikely as this condition is typically exacerbated by steroid use, and our case showed resolution in both eyes after local steroid injection. The development of submacular fibrinoid material in both eyes is unique and may be underrecognized, as OCT may not be commonly obtained in the immediate postoperative period. While subretinal fibrin has been described in CSC,^10^ it is not a recognized feature of diabetic retinopathy.

Although periocular corticosteroid therapy has been associated with reduced incidence of choroidal effusion following diabetic vitrectomy with endolaser,^8^ it is unclear if the triamcinolone acetonide injections led to resolution of the fibrinoid reaction, or if the same outcome would have been achieved with standard postoperative topical corticosteroid, as reported in the case series by Luo et al. Notably, the right eye developed this fibrinoid reaction despite intraoperative posterior subtenon's triamcinolone acetonide injection, suggesting the treatment was not enough to prevent the fibrinoid reaction from happening, but may have had a dampening effect. The left eye, which did not receive an intraoperative corticosteroid injection, developed both a subretinal and transvitreal fibrinoid reaction. Perhaps a milder fibrinoid reaction would have been noted if a corticosteroid injection had been given intraoperatively, but this is uncertain.

## 4 Conclusions

Infectious endophthalmitis should be considered in eyes with significant postoperative inflammation. Early postoperative OCT should be considered following diabetic vitrectomy in patients with significant postoperative inflammatory signs to help identify the presence of subretinal fibrinoid material. Future studies may provide insight into the possible role of endolaser in the development of post-vitrectomy fibrinoid reactions in eyes with evident ischemia and intraoperative corticosteroid injection in preventing or dampening this rare reaction.

## Patient consent

As the images included in this manuscript are entirely anonymized and do not allow for identification of the patient whose clinical course is described, formal patient consent is not required per Elsevier policy.

## Funding

No funding or grant support.

## Authorship

All authors attest that they meet the current ICMJE criteria for Authorship.

## Declaration of competing interest

The following authors have no financial disclosures: Andrew J. Nelson, Neesurg S Mehta, Jorge R. Ochoa, Kareem Moussa.

C.L. is a consultant for Alimera, Regeneron, and Allergan.
